# Metastatic Urothelial Carcinoma: Have We Take the Road to the Personalized Medicine?

**DOI:** 10.3390/cells11101614

**Published:** 2022-05-11

**Authors:** Marco Audisio, Consuelo Buttigliero, Fabio Turco, Marco Donatello Delcuratolo, Chiara Pisano, Elena Parlagreco, Rosario Francesco Di Stefano, Lavinia Di Prima, Veronica Crespi, Giovanni Farinea, Massimiliano Cani, Marcello Tucci

**Affiliations:** 1 Department of Oncology, University of Turin, San Luigi Gonzaga Hospital, 10124 Turin, Italy; consuelo.buttigliero@unito.it (C.B.); turcofabio9@gmail.com (F.T.); donatello.m.delcuratolo@gmail.com (M.D.D.); chiara.pisano1991@gmail.com (C.P.); elena.parlagreco@edu.unito.it (E.P.); rosario-distefano@virgilio.it (R.F.D.S.); lavinia.diprima@libero.it (L.D.P.); veronicacrespi92@gmail.com (V.C.); gio.farinea@gmail.com (G.F.); massi.cani@gmail.com (M.C.); 2 Department of Medical Oncology, Cardinal Massaia Hospital, 14100 Asti, Italy; marcello.tucci@gmail.com

**Keywords:** urothelial cancer, immunotherapy, FGFR inhibitors, enfortumab vedotin

## 1. Introduction

Urothelial cancer is a lethal malignancy characterized by a wide diffusion in Western countries due to a larger exposure to known risk factors, such as aromatic amines, tobacco smoke and benzene [1]. Metastatic disease has a worse prognosis compared with non-muscle invasive disease, with a 5-year overall survival (OS) of 6% versus 90% [2,3].

Non-metastatic urothelial carcinoma treatment is based on tumor excision, often associated with adjuvant local chemotherapy or Bacille Calmette-Guerin (BCG) instillation, depending on relapse risk [4]. Early radical cystectomy, associated with neoadjuvant or adjuvant cisplatin-based chemotherapy, is the standard treatment of muscle-invasive bladder cancer (MIBC) [5]. In cisplatin eligible patients, the most used chemotherapy regimens as first-line treatment are the combination of methotrexate, vinblastine, doxorubicin and cisplatin (MVAC), even in a dose-dense regimen, or the more tolerable association of cisplatin and gemcitabine. [6,7,8] In cisplatin-ineligible patients, treatment options include the combination of carboplatin and gemcitabine and, for those who are platinum ineligible, immunotherapy with pembrolizumab or atezolizumab [9,10,11].

The prognosis of metastatic urothelial carcinoma patients treated with only chemotherapeutic agents is poor. Even in those patients progressing to first-line chemotherapy and eligible for a second-line chemotherapy, the median OS is still 14–16 months [12,13].

Recently, a more accurate understanding of the mechanisms underlying urothelial cancer pathogenesis are leading to the introduction of target therapies showing exciting results in several phase III trials. The approval of immune checkpoints inhibitors (ICIs) and target therapies including fibroblastic growth factor receptor (FGFR) inhibitors or antibody drug conjugates against the nectin-4 has widely changed the treatment landscape of metastatic urothelial carcinoma, demonstrating better activity in terms of outcome compared to chemotherapeutic agents as second and subsequent-line treatment and as maintenance treatment after first-line chemotherapy [14,15,16,17].

## 2. Immunotherapy in Urothelial Cancer: Biological Rationale and Clinical Evidences

Urothelial cancer has some biological features that make it an excellent target for immunotherapy. It harbors high somatic mutation rate, high PD-L1 levels and high T CD8(+) lymphocytes density, suggesting tumor-associated tolerance [18,19,20]. The first immunotherapeutic agent showing significant efficacy in bladder cancer was BCG, a live-attenuated strain of *Mycobacterium bovis* developed in 1921 as a vaccine for tuberculosis that, in 1976, used by intravesical instillation, demonstrated to decrease the rate of recurrence and progression of patients with in situ carcinoma (CIS) or superficial bladder tumors [21]. BCG internalization by urothelial cells and bladder cancer cells induces the secretion of cytokines and chemokines and a massive migration of inflammatory cells into the bladder mucosa and lumen, leading to an immune response against tumor cells. Immune system cell subsets that have potential roles in BCG therapy include CD4(+) and CD8(+) lymphocytes, natural killer cells, granulocytes, macrophages and dendritic cells. Bladder cancer cells are killed by direct cytotoxic activity and by the secretion of soluble factors such as TRAIL (tumor-necrosis-factor-related apoptosis-inducing ligand) [22]. Subsequently several unsuccessful attempts using activating interleukin(IL)-2 and interferon (IFN)-alfa-2B have been made to stimulate the activity of T-lymphocytes against urothelial carcinoma cells both in localized and advanced disease [23].

A revolution in cancer treatment happened with the understanding that cancer cells are able to evade the anti-tumor response through some crucial immune checkpoint pathways, creating a tumor microenvironment interfering with immune system function [24]. A new class of drugs, named ICIs, able to reduce the inhibitory activity of immune checkpoints, was developed.

The most targeted immune checkpoint is the programmed death protein 1 (PD-1)—PD-L1 signaling pathway. PD-1 is a receptor expressed on CD4(+) and CD8(+) T lymphocytes, as well as on several immune cells. PD-L1 and programmed cell death ligand protein-2 (PD-L2) are ligands of PD-1, which can be expressed by tumor cells [25]. The interaction between PD-1 and PD-L1 favors immune system function inhibition and consequently tumor proliferation. Antibodies interfering with PD-1 or PD-L1 signaling are able to increase lymphocytes activity against tumor cells [24]. Another crucial immune checkpoint is Cytotoxic T-lymphocyte-associated antigen 4 (CTLA-4). It is a membrane receptor that act as a negative regulator of T cell responses through interaction with its ligands, CD80 (B7-1) and CD86 (B7-2), expressed on the surface of antigen-presenting cells [25]. This checkpoint was the first to be targeted, with the development of two anti-CTLA-4 antibodies: ipilimumab and tremelimumab.

The contribution of these antibodies to immune activation and the subsequent clinical efficacy were demonstrated in several studies enrolling multiple type of cancers, including urothelial cancer [26,27].

The introduction of ICIs has revolutionized the therapeutic landscape of urothelial cancer. The standard first-line treatment of urothelial cancer patients remains platinum-based chemotherapy. ICIs, in fact, have still not demonstrated better outcomes than chemotherapy in first-line setting.

The PD-1 inhibitor pembrolizumab was tested in a first-line setting in patients with advanced or metastatic urothelial cancer without previous exposure to platinum-chemotherapy firstly as monotherapy in the phase II KEYNOTE-052 trial, showing a promising overall response rate (ORR) of 28.6%. Unfortunately, the phase III KEYNOTE-361 study testing the drug activity as monotherapy or in combination with chemotherapy failed to demonstrate a better progression-free survival (PFS) and OS than chemotherapy [10,28]. Even the association of the CTLA-4 inhibitor tremelimumab and the PD-L1 inhibitor durvalumab in the phase III DANUBE trial did not show better outcome than chemotherapy as first-line treatment [29]. The PD-L1 inhibitor atezolizumab was tested in platinum-naive patients in a cohort of the Phase II single arm IMvigor210 study, showing a promising response rate of 23.5% [11,30]. The most relevant study investigating atezolizumab in a first-line setting in patients with advanced or metastatic urothelial cancer is the IMvigor130 trial, a multicenter, phase III randomized trial testing atezolizumab as monotherapy or in combination with chemotherapy versus chemotherapy alone. Preliminary results are promising, and the trial follow-up phase is ongoing [31].

ICIs were also tested as a maintenance therapy in patients achieving a response or a stability of disease after first-line platinum-based chemotherapy. In the phase III JAVELIN Bladder 100 trial, the anti-PD-L1 antibody avelumab as maintenance therapy in patients with response or stability of disease after a first-line platinum-based chemotherapy achieved the primary endpoint of OS compared to placebo (21.4 vs. 14.3 months) [32]. On the basis of these results, avelumab received Food and Drug Administration (FDA) and European Medicines Agency (EMA) approval in this setting.

As second-line treatment after failure of first-line chemotherapy, pembrolizumab represents now the standard of care, on the basis of the results of the KEYNOTE 045 trial. In this study, that enrolled 542 advanced urothelial carcinoma patients progressing to first-line platinum-based chemotherapy, the median OS was 3 months longer in the pembrolizumab group compared to the chemotherapy group (10.3 vs. 7.4 months) [33]. The EMA granted approval for this drug as therapy of advanced urothelial carcinoma patients progressing to platinum-based chemotherapy or not eligible for cisplatin-based chemotherapy.

Atezolizumab showed promising results as second-line treatment in a cohort of the phase II trial IMvigor210, but the phase III IMvigor211 trial failed to demonstrate an advantage in terms of OS compared to chemotherapy in patients progressing to platinum-based first-line chemotherapy [34,35]. Nivolumab was tested in second-line therapy after platinum-based chemotherapy failure in the single arm phase II Checkmate 275 trial showing a promising response rate [36].

## 3. The FGFR Pathway: A Crucial Actor in Urothelial Carcinoma Pathogenesis

The binding of fibroblast growth factor (FGF) ligands to FGFRs, a group of transmembrane tyrosine kinases receptors, induce the activation of downstream transduction intracellular signaling pathways, including phospholipase C (PLC)γ, phosphatidylinositol 3-kinase (PI3K) -AKT, and RAS- mitogen-activated protein kinase (MAPK) pathways involved in tumoral cells differentiation and growth [37,38,39]. FGFR pathway function alterations are implicated in carcinogenesis processes. In recent years, several FGFR genomic aberrations have been discovered across several types of cancers. Urothelial carcinoma, cholangiocarcinoma and endometrial cancer are those with the highest frequency of FGFR alterations [40,41].

FGFR3 is the most frequently hyperactivated FGF-Receptor in urothelial cancer. 60% of early and 20% of advanced bladder cancer are characterized by hyperactivation of this tyrosine kinase receptor [39,42,43].

The understanding of the crucial function of FGFR signaling aberrations in oncogenesis led to the development of multiple agents able to interfere with this pathway [38].

The FGFR1-3 selective oral inhibitor infigratinib showed promising results in terms of response rate (25–38%) and disease control rate (64–75%) in two phase I trials enrolling advanced urothelial cancer patients [44,45]. A phase III clinical trial is currently ongoing investigating the activity of this agent as adjuvant treatment following cystectomy in bladder cancer patients with targetable FGFR3 aberrations.

Erdafitinib is an all-FGFR oral inhibitor showing a response rate of 40% in a phase II trial enrolling advanced urothelial patients progressing to first-line platinum-based chemotherapy [46]. On the basis of these results, FDA granted accelerated approval of this agent as second-line therapy in urothelial cancer and a phase III trial testing erdafitinib versus pembrolizumab or chemotherapy is currently ongoing.

The FGFR1-3 inhibitor AZD4547 showed promising response rate in a phase II trial in 48 patients with different cancers harboring FGFR alterations [47].

Rogaratinib is a potent FGFR 1-4 inhibitor firstly tested in a phase I trial enrolling patients with different types of advanced cancers demonstrating promising activity and an acceptable safety profile. A phase II-III trial showed that this agent has similar efficacy compared to standard chemotherapy [48,49].

The FGFR1-4 inhibitor Pemigatinib was successfully tested in a phase II trial enrolling solid tumors with FGFR alterations achieving promising results in the cohort of patients with cholangiocarcinoma [50]. The results in the urothelial cancer cohort are pending.

## 4. The Nectin-4 Targeting Antibody-Drug Conjugate: An Innovative Vision of Carcinoma Treatment

Nectin-4 is a transmembrane polypeptide member of the nectin family encoded by the gene NECTIN4 [51]. Aberrant expression of Nectin-4 has been identified in multiple types of cancers, such as bladder, breast, lung, pancreatic and ovarian cancers. This polypeptide is able to activate the PI3K-AKT molecular pathway [52,53,54]. Urothelial carcinoma is one of the tumors with the highest nectin-4 expression. Nearly 80% of urothelial carcinoma patients have some levels of expression, and 30% have a strong expression of Nectin-4 [52].

Antibody-drug conjugates (ADCs) are monoclonal antibodies associated with cytotoxic agents. ADCs are directed against overexpressed tumor-associated antigens and their innovative mechanism of action couples the cytotoxic payload activity with the precision of the antibody. ADCs in fact reduce the exposure of normal tissues to the cytotoxic agent decreasing potential toxicities [55]. When an ADC binds to its cellular target (antigen), the ADC–antigen complex is internalized, and the payload is released in the intracellular compartment to trigger cytotoxicity [16,55].

The most used payloads include those able to act on DNA and those interfering with microtubule formation. Monomethyl auristatin-E (MMAE) is the most used microtubule-acting bystander. Indeed, this membrane’s permeability allows the diffusion from antigen-positive tumor cells into neighboring cells killing them in an antigen-independent manner, obtaining what is known as the “bystander” killing effect [16,55].

Nectin-4 represents an ideal target for an ADC in urothelial carcinoma because of its high expression in cancer cells and its low expression in normal tissues. Enfortumab vedotin is an ADC composed by a monoclonal antibody against Nectin-4 attached to the payload MMAE [56]. After promising results in phase I trials [57,58], in the phase II single arm EV-201 enfortumab vedotin demonstrated an interesting ORR of 52% as second-line treatment after ICIs in platinum ineligible disease and of 44% as third-line chemotherapy and immunotherapy [59,60]. On the basis of these evidences, in 2019, the FDA granted accelerated approval for this agent in patients progressing after platinum-based chemotherapy and immunotherapy [61]. Subsequently, the phase III EV-301 trial demonstrated a statistically significant improvement of 4 months (12.8 versus 8.9 months) compared to physician’s choice chemotherapy in advanced urothelial cancer patients progressing to platinum-based chemotherapy and immunotherapy [62].

Multiple trials testing enfortumab vedotin in urothelial cancer patients are currently ongoing. The EV-302 trial is a phase III randomized controlled study testing the association of enfortumab vedotin and pembrolizumab in first-line therapy compared to platinum-based chemotherapy, and the EV-103 trial is a multi-cohort phase Ib/II study testing enfortumab vedotin in combination with pembrolizumab and/or chemotherapy or alone in advanced or metastatic urothelial cancer patients (NCT03288545). Preliminary results of the trial cohort testing the association of enfortumab vedotin and pembrolizumab in cisplatin-ineligible untreated patients with advanced urothelial carcinoma were presented at the ASCO 2021 annual meeting, and the ORR was 73.3% (95% CI: 58.1, 85.4) [63].

## 5. Molecular Characterization: The New Challenge in Urothelial Cancer Treatment

Urothelial cancer is a molecularly heterogeneous disease. In the last years, genomic sequencing led to the classification of urothelial carcinoma into different molecular subtypes, aiming for a more accurate prediction of tumor prognosis and response to therapeutic agents [64]. Initially, four molecular subtypes were identified [65]. Transcriptomic profiles studies led in 2020 to a Consensus Molecular Classification including six molecular clusters.

The Luminal Papillary (LumP) harboring often FGFR aberrations accounts for nearly 24% of urothelial carcinoma and is associated with lower stage disease.

The Luminal Unstable (LumU) accounts for around 15% of cases, showing a wide enrichment of copy number variations and genomic instability. The highest expression of tumor protein (TP)53 and ERCC2 mutations shown in this subtype is the reason of the higher sensitivity to chemotherapy.

The Luminal Non-Specified (LumNS) (8%) is associated with carcinoma in situ and histological micropapillary carcinoma.

The basal/squamous (Ba/Sq) subtype tumors accounts for around 35% of cases showing high basal cell marker genes expression and luminal cell marker genes loss. The most frequently mutated genes were retinoblastoma Protein 1 (RB1) and TP53. This subtype has a poor prognosis, often found in female patients and presented at higher stage.

The stroma-rich (15%) subtype is associated with stromal cell infiltration and high expression of fibroblast and myofibroblast, smooth muscle and endothelial gene signatures.

The Neuroendocrine-Like (NE-like) subtype accounts for nearly 3% of urothelial carcinomas and is the most aggressive subtype showing neuroendocrine histological features and high proliferation rate [43,66].

The molecular clusters associated with better outcome are LumP, LumNS and stromal-rich tumors; LumU is associated with intermediate prognosis, Ba/Sq and NE-Like tumors are characterized by the worst prognosis [43].

The identified molecular subtypes could benefit differently of the therapeutic agents available. NE-like and LumU tumors have molecular features associated with elevated cell proliferation, suggesting a potential response to radiotherapy and chemotherapy [67]. The LumP subtype is associated with FGFR aberrations, allowing the use of agents able to interfere with FGFR. Ba/Sq tumors are characterized by high expression of epidermal growth factor receptor suggesting sensitivity to targeted therapies against this pathway. In addition, Ba/Sq tumors might be more responsive to ICIs, due to the high expression of antigen-presenting machinery genes [43].

## 6. Conclusions

Recently the treatment landscape of metastatic urothelial carcinoma patients has been revolutionized by the introduction of innovative agents.

Platinum-based chemotherapy remains the backbone of first-line therapy while immunotherapy is now the standard of care in second-line setting after failure of first-line chemotherapy, as maintenance therapy in patients responding to first-line chemotherapy and in first-line in cisplatin-ineligible PD-L1 positive patients or in those not eligible for any platinum-containing chemotherapy [32,33,68,69]. In patients progressing on ICIs, FGFR inhibitors or enfortumab vedotin are available agents on the basis of FGFR status. In addition, patients with progressive disease after chemotherapy not already treated with immunotherapy, can be treated with erdafitinib if FGFR-positive or, if negative, with ICIs.

The new molecular classification of urothelial carcinoma in six subtypes with different prognosis and therapy response is paving the way for the tailored treatment of urothelial carcinoma patients. A new era is opening up also in urothelial carcinoma, and we will be able soon to treat each patient on the basis of the molecular characteristics of disease.
